# Immunosuppressive Therapy in Takayasu Arteritis: A Tightrope Walk Between Inflammation and Infection

**DOI:** 10.7759/cureus.97954

**Published:** 2025-11-27

**Authors:** Somnath Panda, Subramanian Narayanan Kaladi, Tushar B Jagzape

**Affiliations:** 1 Internal Medicine, All India Institute of Medical Sciences, Raipur, Raipur, IND; 2 Pediatrics, All India Institute of Medical Sciences, Raipur, Raipur, IND

**Keywords:** adalimumab, immunosuppressive therapy, rheumatological disorders, takayasu arteritis (ta), tuberculous meningitis (tbm)

## Abstract

Infectious diseases like tuberculosis (TB) are highly prevalent in developing countries. Rheumatological diseases are also not uncommon. Both are chronic illnesses with overlapping symptoms and may present a diagnostic challenge. Associations between TB and Takayasu arteritis (TA) have been reported. Immunosuppressive therapy for rheumatological disease always poses a risk of reactivation of infections like TB. Here we report a case of a 12-year-old Indian male child, a known case of TA with bilateral renal artery stenosis on methotrexate, adalimumab, and multiple antihypertensives, who presented with eight days of fever, cough, vomiting, and backache. Initially, the diagnosis of infectious etiology versus disease reactivation was considered. Although on evaluation, septic foci were not identified, and subsequently, pulse methylprednisolone was started. There was some initial improvement, but then the patient deteriorated with a severe headache followed by seizures, altered sensorium, and features of raised intracranial pressure (ICP). Imaging revealed features suggestive of tuberculous meningitis (TBM) with obstructive hydrocephalus, but the cartridge-based nucleic acid amplification test (CBNAAT) was negative in both endotracheal (ET) aspirate and CSF aspirate, leading to a clinico-radiological diagnosis of TBM. He underwent ventriculoperitoneal (VP) shunting and was started on anti-tubercular therapy (ATT). This case emphasizes the need for regular vigilance for infectious risk in rheumatological diseased pediatric populations who are chronically immunosuppressed.

## Introduction

Takayasu arteritis (TA) is a chronic vasculitis that primarily affects the aorta and its major branches, specifically the large vessels. Cases are seen worldwide, but the distribution is variable. As compared to Europe and North America, it is more common in South America, Africa, and Asia. Though recognized as a common cause of renovascular hypertension in Asian children, the exact prevalence data are not available. The reported prevalence varies from 40 per million to 0.9 per million in Japan and the United States, respectively [1]. The chronic inflammation leads to vascular stenosis, ischemia, and end-organ damage. It predominantly occurs in young females, with childhood-onset cases being even rarer. The initial clinical presentations are nonspecific and include fever, myalgia, malaise, weight loss, pain in the abdomen, joint pain, etc., in the early phase. In the later phase, the symptoms are dominated by the vessel affected and include heart failure, neurological events, or raised blood pressure [1,2]. The mainstay of treatment is the use of immunosuppressants like steroids, mycophenolate mofetil (MMF), azathioprine, and biologicals like tumor necrosis factor (TNF)-alpha inhibitors [2]. In a developing country like India, where the burden due to infectious diseases is high, continued use can increase susceptibility to the prevalent infections, including tuberculosis (TB). According to the World Health Organization (WHO), in 2023, India bore a significant TB burden, with an estimated 2.8 million incident cases (195 per 100,000 population) [3]. Although TB primarily affects the lungs, it can disseminate to any organ system in the body [4], with extrapulmonary TB, like lymph node and central nervous system TB, being more common in children in comparison to adults. Tuberculous meningitis (TBM) remains a major cause of neurological morbidity in endemic regions, particularly in immunosuppressed individuals. Due to its insidious onset and overlapping symptoms with other inflammatory or infectious causes, the diagnosis is often delayed, making the prognosis worse [5]. This case emphasizes the importance of maintaining a precarious balance between therapeutic immunosuppression and the risk of infections in managing rheumatological diseases.

## Case presentation

A developmentally normal 12-year-old male child presented to the emergency department with an eight-day history of fever, cough, vomiting, and back pain. The fever was acute in onset, progressively worsening, and documented at three spikes per day, relieved temporarily with medication. The cough was dry and not associated with chest retractions or respiratory distress. Vomiting occurred one to two times per day, was non-projectile, and was not blood-tinged. The child also reported backache without any preceding trauma, localized swelling, or neurological symptoms. The child was diagnosed with TA in 2021 when he presented with a history of prolonged fever without any localizing symptoms. He was found to have poor pulses in both lower limbs and hypertension in the upper limbs. Subsequently, on investigation, he was found to have arteritis changes involving both renal arteries and the abdominal aorta. He was started on steroids and methotrexate. In view of persistent and uncontrolled hypertension, he underwent bilateral renal artery balloon angioplasty for renal artery stenosis in 2024. He was on multiple antihypertensive medications (prazosin, amlodipine, clonidine, hydrochlorothiazide, and labetalol) and immunosuppressive therapy, including methotrexate and adalimumab, which was added subsequently. There was no reported history of TB exposure, and he had received the Bacillus Calmette-Guérin (BCG) vaccine in childhood.

On presentation, the child was alert and hemodynamically stable. The child was afebrile with a pulse rate of 94 beats per minute, a respiratory rate of 26 breaths per minute, and blood pressure readings of 128/86 mmHg in the right arm (~95-98th percentile systolic, >95th percentile diastolic) and 124/76 mmHg in the left arm (~90-95th percentile systolic, ~75-90th percentile diastolic). The child had pallor but no icterus, cyanosis, or lymphadenopathy. Systemic examination revealed no murmur on cardiac auscultation, normal breath sounds bilaterally, and a soft, non-tender abdomen without organomegaly. Neurologically, the child was alert with normal tone and reflexes and no signs of meningeal irritation or raised intracranial pressure​ (ICP). Given the persistent fever and history of immunosuppression, an infectious etiology versus disease reactivation was suspected. Initial blood work showed anemia, leukocytosis with neutrophilic predominance, and elevated inflammatory markers (Table 1).

**Table 1:** Laboratory parameters of the patient CRP: C-reactive protein; ESR: erythrocyte sedimentation rate

Parameter	Patient Value	Reference Range	Interpretation	Time Point
Hemoglobin	9.6 g/dL	11.5–15.5 g/dL	Decreased	Admission
Total Leukocyte Count	18,600	4,000–11,000 /mm³	Increased	Admission
Neutrophils (%)	82.8%	40–75%	Increased	Admission
CRP	73.77 mg/L	<5 mg/L	Increased	Admission
ESR (1 hr)	70 mm/hr	<20 mm/hr	Increased	Admission
CSF Protein	60.4 mg/dL	15–45 mg/dL	Increased	Post-neuro symptoms
CSF Glucose	45 mg/dL	>2/3 of serum glucose	Decreased	Post-neuro symptoms

**Figure 1:**
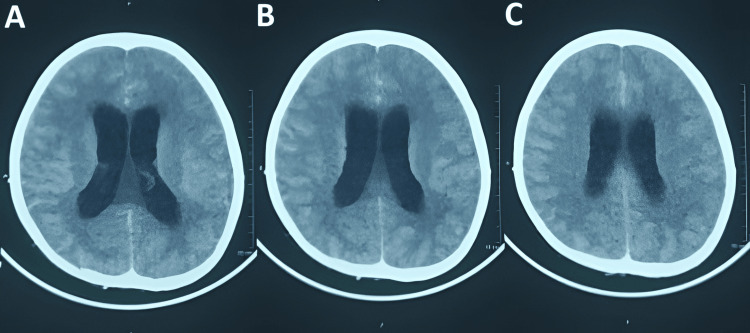
Non-contrast computerized tomography of the head A: axial image at the level of the basal ganglia showing gross dilatation of the bilateral lateral ventricles with periventricular hypodensity; B, C: higher axial sections showing continued ventricular dilatation with periventricular low attenuation consistent with CSF ooze.

However, blood and urine cultures were negative. Due to continued fever, antibiotic therapy was escalated to piperacillin-tazobactam. When fever persisted despite antibiotic treatment, the possibility of a TA flare/reactivation was considered, and a short course of methylprednisolone was administered. There was only a transient improvement in the symptoms. Subsequently, the child developed neurological symptoms, including abnormal sensations described as "insect crawling" and a headache localized bilaterally to the frontal region, increased muscle tightness, and a decline in motor strength to 3/5 in all four limbs. The child further had an episode of posturing, which was aborted with midazolam, and levetiracetam was started. CSF analysis demonstrated lymphocytic pleocytosis, elevated protein, and low glucose (Table 1). Acid-fast bacilli (AFB) and cartridge-based nucleic acid amplification test (CBNAAT) testing from endotracheal (ET) secretions and CSF were negative. Non-contrast computerized tomography (NCCT) of the head, as shown in Figure 1, showed non-obstructing hydrocephalus, and fundoscopy confirmed grade IV papilledema.

**Figure 2:**
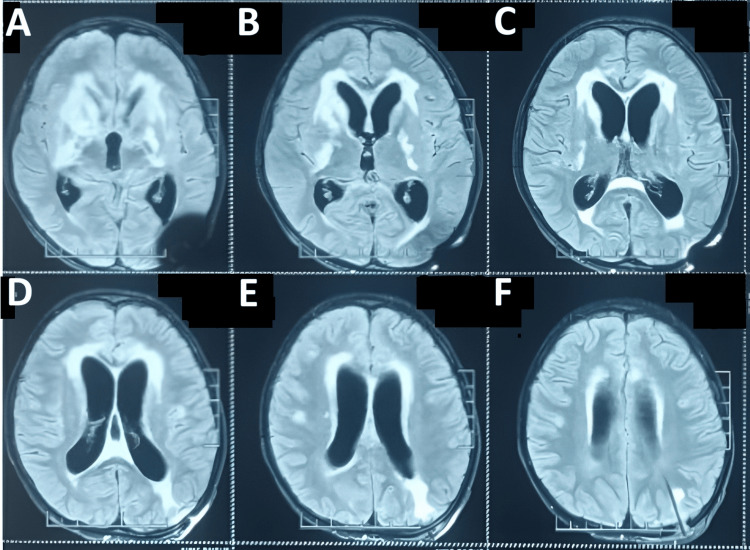
MRI of the head (plain) A: axial section at the level of the basal ganglia showing a burr-hole defect in the left parietal bone; B: axial section at the level of the midbrain and thalamus showing dilatation of the lateral ventricles and periventricular hyperintense signal changes suggestive of transependymal CSF seepage; C: axial section through the body of the lateral ventricles, demonstrating moderate dilatation of both lateral and third ventricles; D: higher axial cut showing ventricular dilatation with periventricular hyperintensity indicating interstitial edema; E: axial section at the level of centrum semiovale showing prominent lateral ventricles with surrounding hyperintensities along the ventricular margins; F: axial section at the level of corona radiata demonstrating enlarged lateral ventricles and diffuse periventricular hyperintensity consistent with transependymal CSF seepage.

Hydrocephalus was further confirmed by MRI, as shown in Figure 2.

**Figure 3:**
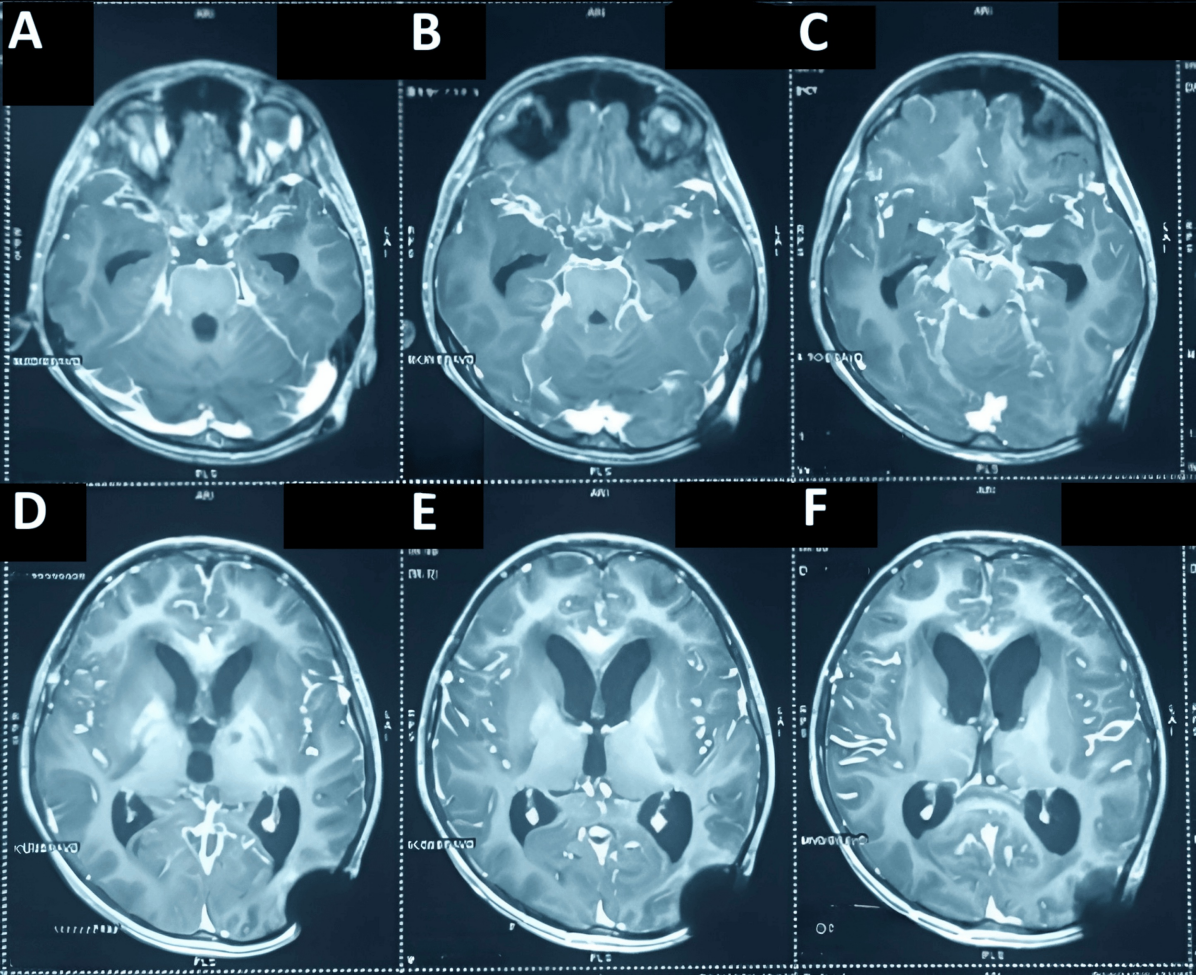
MRI of the head post-contrast axial T1-weighted MRI (T1 + gadolinium) A: section at the level of the prepontine cistern and cerebellum showing leptomeningeal enhancement along the basal cisterns; B, C: section at the level of the midbrain and thalami depicting enhancement of the basal meninges and suprasellar cisterns; D: section through the lateral ventricles showing the ventriculoperitoneal (VP) shunt tip in the left lateral ventricle with mild ventricular prominence; E: a higher section showing enhancement along cortical sulci and basal cisterns that is consistent with meningitis; F: section through centrum semiovale showing minimal leptomeningeal enhancement and the parietal burr hole site on the left corresponding to VP shunt placement.

Further MRI with contrast was done, and the findings, as shown in Figure 3, were suggestive of tubercular meningitis.

An extensive search for TB foci was carried out, including chest X-ray, CT abdomen, and purified protein derivative/tuberculin skin test (PPD/TST), all of which turned out to be negative. The reason could have been due to immunosuppressive therapy. Because of financial concerns, the interferon gamma release assay (IGRA) was not repeated. IGRA, which was done before starting adalimumab in the past, was negative. Considering the clinical picture and neuroimaging findings, a ventriculoperitoneal (VP) shunt was inserted, and anti-tubercular therapy (ATT) with isoniazid (5 mg/kg OD), rifampicin (10 mg/kg OD), pyrazinamide (25 mg/kg OD), ethambutol (15 mg/kg OD), and pyridoxine (10 mg OD) was initiated. Adjunctive prednisolone (2 mg/kg/day) was administered to reduce cerebral edema. During hospitalization, the child had multiple extubation failures necessitating a tracheostomy. The child later developed dystonia, which was managed pharmacologically with tetrabenazine, levodopa-carbidopa, and trihexyphenidyl, and urinary retention requiring intermittent catheterization. The hospital course was further complicated by a VP shunt blockage requiring revision surgery and a superimposed *Klebsiella pneumoniae* tracheal infection. The child remained hypertensive, requiring adjustment of antihypertensive therapy. The child was gradually weaned off the ventilatory support. Parents were trained regarding the care of tracheostomy and home management of seizures. After the parents were confident, the child was discharged after 40 days of admission with a plan to continue ATT and antihypertensive therapy. The child was followed up regularly in the OPD. In the last follow-up, the child was stable with blood pressure between the 50th to 90th centiles with no fresh seizure episodes, but unfortunately, the child is left with various neurological sequelae in the form of hypertonia and dystonia of all four limbs with bulbar palsy requiring tube feeding.

## Discussion

The association between TA and TB has been extensively explored in the literature. Multiple studies have suggested a potential etiological link between *Mycobacterium* tuberculosis and the pathogenesis of TA. Given the histopathological similarities of panarteritis and giant cell granulomatous reactions in both conditions, hypotheses range from immune cross-reactivity between mycobacterial antigens and human heat shock proteins to direct vascular invasion by *Mycobacterium* tuberculosis [6,7].

TNF plays a critical role in maintaining granuloma integrity, which is essential for controlling latent TB infection. TNF blockade disrupts these granulomas, allowing *Mycobacterium* tuberculosis to proliferate. Studies have reported up to 25 times higher risk of TB reactivation by the use of TNF-alpha inhibitors such as adalimumab in TA management [8,9]. Peng et al. highlighted the importance of pre-treatment TB screening due to an increased incidence of TB in TA patients [10]. Furthermore, retrospective analyses have highlighted the high prevalence of latent TB in TA patients [11,12]. Hence, screening for latent TB should be a prerequisite before initiating immunosuppressive therapy in TA patients. The use of IGRA and chest imaging is advocated for the same, although these tests have shown limitations in immunocompromised patients [10]. In our case, TB reactivation could have likely been attributed to the use of methotrexate and adalimumab; however, our patient had gone through PPD testing, IGRA, and CXR before receiving methotrexate and adalimumab, and the results came out to be negative.

There was also a lack of prior TB exposure history, and he had received the BCG vaccination. Previous case reports have primarily documented TB reactivation affecting the lungs, lymph nodes (tuberculous lymphadenitis), and large vessels (TB aortitis), among other sites, particularly in patients with TA receiving TNF inhibitors [8,13]. However, to our knowledge, TBM has been rarely reported in this context, especially in pediatric TA patients.

The diagnostic challenge of TBM is further compounded when microbiological tests, including AFB smear and CBNAAT, yield negative results. CBNAAT has a low sensitivity for CSF samples, with a reported range from 40% to 80% [14]. In our case, despite high clinical suspicion, microbiological confirmation was lacking, necessitating reliance on radiological and clinical assessment to establish the diagnosis [15,16]. Corticosteroids are a mainstay in both TA and TBM treatment; however, their immunosuppressive effects can obscure TB symptoms, leading to delayed diagnosis and treatment initiation [8]. Immunosuppressive agents such as methotrexate, cyclophosphamide, and biologics have been employed in refractory TA cases, yet their use must be cautiously balanced against TB risk [17]. In our case, the child was initially treated with pulse methylprednisolone for suspected TA reactivation, but lack of improvement and neurological deterioration prompted further evaluation for a neurological cause. Following the clinico-radiological diagnosis of TBM, immunosuppressants were adjusted, with methotrexate and adalimumab being withheld. TBM frequently leads to complications such as hydrocephalus, which occurs in a significant proportion of patients, and severe neurological sequelae, including cognitive impairment, seizures, and neurogenic bladder dysfunction, some of which may be irreversible despite TB clearance [15,18]. Our patient developed multiple TBM-related complications, including hydrocephalus requiring a surgical intervention in the form of a VP shunt, dystonia, neurogenic breathing necessitating tracheostomy, and urinary retention. The need for prolonged intensive care, repeated shunt revisions, and extensive rehabilitative support highlights the severe neurological burden of TBM in immunosuppressed patients.

The interplay between immunosuppressants and anti-TB treatment requires careful modulation to prevent both disease relapse and opportunistic infections. Current literature suggests that monitoring clinical signs of TB reactivation may be more reliable than IGRA alone. Annual TB screening is recommended for patients on long-term immunosuppressants, especially TNF inhibitors, with consideration given to prophylactic TB treatment in high-risk cases [19].

## Conclusions

Immunosuppressive therapy in TA requires careful risk assessment, particularly in TB-endemic regions, due to the heightened susceptibility to reactivation of latent disease. Their initial presentation can mimic disease relapse, making differentiation difficult. Pre-treatment screening and prophylactic therapy for latent TB are essential in patients requiring prolonged immunosuppressive therapy. TB reactivation in TA can virtually affect any organ system, with TBM being one of the rarer yet severe manifestations, especially in pediatric cases. TB meningitis may require a clinico-radiological diagnosis when microbiological tests like AFB smear and CBNAAT are negative, ensuring timely management and improving overall outcomes in immunosuppressed patients.
